# The breast cancer microenvironment and lipoprotein lipase: Another negative notch for a beneficial enzyme?

**DOI:** 10.1002/2211-5463.13559

**Published:** 2023-01-30

**Authors:** Makayla M. Bavis, Allison M. Nicholas, Alexandria J. Tobin, Sherri L. Christian, Robert J. Brown

**Affiliations:** ^1^ Department of Biochemistry Memorial University of Newfoundland St. John's NL Canada

**Keywords:** breast cancer, estrogen receptor, human epidermal growth factor receptor 2, lipase maturation factor, lipoprotein lipase, progesterone receptor

## Abstract

The energy demand of breast cancers is in part met through the β‐oxidation of exogenous fatty acids. Fatty acids may also be used to aid in cell signaling and toward the construction of new membranes for rapidly proliferating tumor cells. A significant quantity of fatty acids comes from the hydrolysis of lipoprotein triacylglycerols and phospholipids by lipoprotein lipase (LPL). The lipid obtained via LPL in the breast tumor microenvironment may thus promote breast tumor growth and development. In this hypothesis article, we introduce LPL, provide a meta‐analysis of RNAseq data showing that LPL is associated with poor prognosis, and explain how LPL might play a role in breast cancer prognosis over time.

## Graphical abstract

This hypothesis article introduces lipoprotein lipase (LPL), provides a meta‐analysis of *LPL* mRNA expression in breast cancer, and explores a possible role for LPL in breast cancer. Overall, we hypothesize that the lipid hydrolysis products that are generated from lipoproteins by LPL impact the breast cancer cell microenvironment, ultimately decreasing prognosis due to increased proliferation and metastasis.

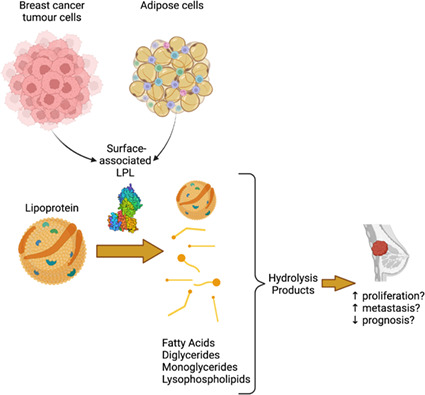

**Abbreviations**
ER: estrogen receptor
GPIHBP1: glycosylphosphatidylinositol‐anchored high‐density lipoprotein binding protein 1
HER2: human epidermal growth factor receptor 2
HR: hazard ratio
IL: interleukin
LMF1: lipase maturation factor 1
LPL: lipoprotein lipase
PR: progesterone receptor
TNF: tumor necrosis factor
VLDL: very low‐density lipoprotein

It is estimated that two in five Canadians will have a cancer diagnosis in their lifetime [1]. Of newly diagnosed cases in Canada, it was projected for 2022 that approximately one in four cases will be breast cancer—representing one in every eight Canadian women [2]. Furthermore, of projected cancer deaths for 2022, approximately one in seven will be deaths due to breast cancer—representing one in every 34 women in Canada [2]. Breast cancer is a heterogeneous type of cancer with different etiologies and pathophysiologies, depending on the subtype. Breast tumors may be basal or luminal (type A or type B), but they also have a basic classification of subtypes based on their expression of three different tissue receptors: estrogen receptor (ER), progesterone receptor (PR), and human epidermal growth receptor 2 (HER2) [3] (Table 1). Of note, the triple‐negative breast cancer subtype, which lacks the expression of all three receptors, is considered highly aggressive, with one of the highest rates of metastasis and the poorest rates of survival out of all breast cancer subtypes [3].

**Table 1:** Basic molecular subtypes of breast cancer.

Subtype	Receptor status		
	Estrogen receptor (ER)	Progesterone receptor (PR)	Human epidermal growth factor receptor 2 (HER2)
Luminal A	+	+/−	−
Luminal B	+	+/−	+
HER2‐enriched	−	−	+
Triple negative	−	−	−

To keep pace with the metabolic demands associated with rapid proliferation, differentiation, and angiogenesis, cancer cells require more fatty acids for the growth and development of tumors [4]. Fatty acids are metabolized to yield acetyl‐CoA through β‐oxidation. The acetyl‐CoA can subsequently feed into the citric acid cycle to produce energy products such as ATP [5]. Aside from their use for energy purposes, select fatty acids can also act as secondary signaling messengers in signaling pathways that serve many functions in maintaining homeostasis within the cell [6]. The dysregulation of signaling pathways in cancer cells, such as the phosphoinositide 3‐kinase/Akt pathway, causes changes in lipid metabolism that can promote cancer cell proliferation, survival, and migration [5]. Furthermore, cancer cells may manipulate the fatty acids incorporated in membranes to alter the membrane fluidity [5], thus changing which molecules can cross membranes to favor cancer cell survival [7]. Therefore, fatty acids are essential to cancer cell development and survival.

Cancer cells exploit two mechanisms for acquiring fatty acids: *de novo* lipogenesis via fatty acid synthase and cytosolic acetyl‐CoA (primarily derived from metabolites that yield cytosolic citrate), and from extracellular lipolysis (Fig. 1) [8, 9]. With normal somatic cells, lipogenesis only occurs in adipocytes and hepatocytes [5]. However, cancer cells tend to exhibit increased fatty acid synthase activity to endogenously supply fatty acids to cancer cells in order to meet the high demand [10]. On the contrary, extracellular lipolysis involves the hydrolysis of lipoprotein triacylglycerols (to liberate *sn*‐1/3 fatty acyl groups) and phospholipids (to liberate the *sn*‐1 fatty acyl group), which can occur via lipoprotein lipase (LPL) (Fig. 1). The resultant free fatty acids may then be taken up by the cells via CD36, the transmembrane channel for cellular fatty acid uptake [11].

**Fig. 1:**
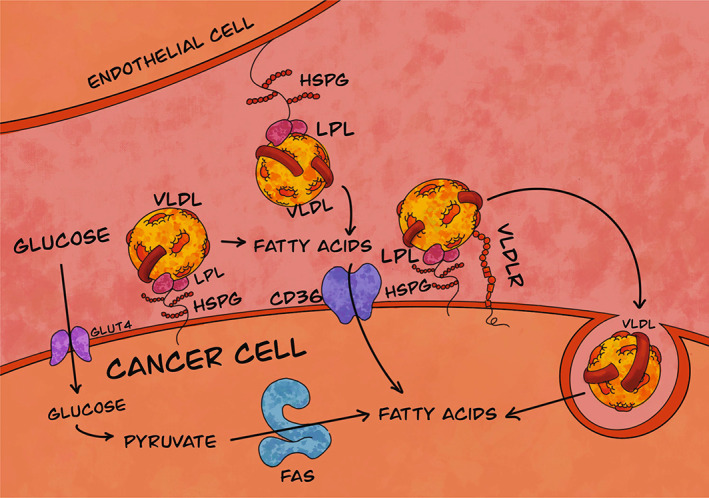
Lipogenesis and lipolysis pathways for fatty acid acquisition by tumor cells. The process of lipolysis involves the exogenous uptake of fatty acids facilitated by lipoprotein lipase (LPL) anchored to heparan sulfate proteoglycans (HSPG). LPL hydrolyzes fatty acids from triacylglycerol‐rich lipoproteins, such as very low‐density lipoproteins (VLDL), and the uptake of fatty acids by the cell is facilitated by the transmembrane protein transporter CD36. LPL also assists in receptor‐mediated endocytosis for intracellular hydrolysis lipids from lipoproteins to yield fatty acids. The process of lipogenesis involves the exogenous uptake of glucose, by glucose transporter 4 (GLUT4), for the *de novo* synthesis of fatty acids from glucose. FAS, fatty acid synthase; VLDLR, very low‐density lipoprotein receptor.

Lipoprotein lipase is an essential enzyme in lipoprotein metabolism that releases free fatty acids from acylglycerols for delivery to cells for various functions. Interestingly, the most detrimental subtype of breast cancer, triple‐negative breast cancer, has been shown to exploit exogenous lipid uptake in part through LPL [12]. Thus, an exploration into the role(s) LPL may play in various breast cancer subtypes is warranted. This hypothesis article aims to introduce LPL, provide a meta‐analysis of *LPL* mRNA expression in breast cancer from data mined via the KMplot online analysis tool [13], and explore its possible role in breast cancer.

## Materials and methods

### KMPlot analyses

A meta‐analysis of RNAseq data for the expression of *LPL* mRNA in human breast cancer tissue was carried out using the kmplot online software (http://www.kmplot.com/) [13]. The datasets examined were primarily from The Cancer Genome Atlas database (personal communication, Balázs Győrffy, Hungarian Academy of Sciences, Budapest, Hungary). Human subjects were defined as high expressors or low expressors of *LPL* mRNA based on the automatic calculation of the upper and lower quartiles of the arbitrary values of RNAseq data. The datasets were examined for all combinations of positive and negative statuses of ER, PR, and HER2; in addition, datasets were examined for the PAM50 basal, luminal A, and luminal B subtypes. Hazard ratios (HR) and statistical analyses were calculated with the online software, as previously described [13].

A meta‐analysis of Affymetrix gene chip microarray data for the expression of *LPL* mRNA in human breast cancer tissue was carried out using the kmplot online software (http://www.kmplot.com/) [13]. Human subjects were defined as high expressors or low expressors of *LPL* mRNA, based on the median of the arbitrary values for the mean expression data of the two available *LPL* probes (205348_s_at and 205349_s_at). Biased microarray data were excluded from analyses. The datasets were examined for all combinations of positive and negative statuses of ER, PR, and HER2. ER status was defined to be both confirmed by immunohistochemistry and microarray. HRs and statistical analyses were calculated with the online software, as previously described [13].

### Lipoprotein lipase activity

MCF‐7, T47D, MDA‐MD‐231, and SKBR3 cell lines were obtained and cultured as previously described [14]. To collect media for LPL activity, cells were initially cultured in 6‐well plates (9.7 × 10^5^ cells per well) for 24 h. After 24 h, cells were incubated in the absence or presence of 1 mL of 100 U·mL^−1^ heparin for 30 min. After 30 min, media were centrifuged at 1000 *g* to pellet any debris, and the supernatant was stored at −80 °C until needed. For positive control, HEK293 cells were transfected with pcDNA3.hHL, and conditioned media without or with heparin were collected, as previously described [15, 16]. To examine LPL activity, 15 μL of media were used with 1,2‐*O*‐dilauryl‐*rac*‐3‐glutaric‐resorufin ester as the substrate, as previously described [17].

### Breast tissue and cell line *LMF1* mRNA expression

Gene expression data from 20 breast cancer cell lines, 31 breast cancer patient samples, and six normal breast tissue samples were obtained from the Gene Expression Omnibus database (GPL570‐Affymetrix Human Genome U133 Plus 2.0 Array platform), as previously described by Pitts et al. [18]. The data were robust multichip average normalized, as previously described [18]. The NetAffx Analysis Center by Affymetrix was used to identify the *LMF1* probe IDs, which were then used to filter and average the gene expression data. Gene expression data were analyzed and grouped by subtype via complete linkage hierarchical cluster analysis using Euclidean distance with genesis 1.8.1 [19].

## Results and Discussion

### LPL protein and expression

Lipoprotein lipase is a glycosylated enzyme that is well‐studied for its roles in lipid metabolism and its negative role in atherosclerosis [20, 21, 22]. The enzyme, originally termed ‘clearing factor’, was first reported based on the study of postprandial dogs receiving heparin to displace the enzyme activity into the bloodstream [23]. Eventually, the gene encoding LPL protein was identified on human chromosome 8p22, spanning a 30 kb region [24]. The gene expression for LPL is influenced transcriptionally by select fatty acids and agonists of the nuclear receptors liver X receptor, retinoic acid X receptor, and peroxisome proliferator‐activated receptors‐α and ‐γ [25].

Until 2018, much of the knowledge about the LPL structure was based on modeling human pancreatic lipase, the structure of which was first elucidated in 1990 [26]. Pancreatic lipase is a family member of the extracellular *sn*‐1 lipase that includes LPL and two other family members, hepatic lipase and endothelial lipase [27]. In 2018, the structure of LPL was solved, complexed as a ligand to one of its extracellular binding partners, glycosylphosphatidylinositol‐anchored high‐density lipoprotein binding protein 1 (GPIHBP1) [28], which is an important protein in the translocation of LPL [29]. Similar to pancreatic lipase, LPL includes two distinct domains: an amino‐terminal α/β‐hydrolase domain and a carboxyl‐terminal polycystin‐1/lipoxygenase/α‐toxin domain [28]. The amino‐terminal domain contains a serine‐aspartate‐histidine charge relay active site. The amino‐terminal domain also contains a ‘lid region’ that covers the active site and is responsible for lipid substrate specificity with the active site [28]. The carboxyl‐terminal domain contains the region responsible for binding to GPIHBP1 and heparan sulfate proteoglycans [28].

Lipoprotein lipase is expressed in several tissues, with the highest levels of expression within the smooth muscle, skeletal muscle, and adipose tissue [30, 31]—cells that require large amounts of fatty acid for energy and storage [32]. Of note, cancer cells undergo lipidomic remodeling and can use fatty acids derived from LPL to accommodate the increased demand of fatty acids for cancer cell growth and proliferation [5].

Once synthesized, LPL is secreted from cells into the subendothelial space to interact with heparan sulfate proteoglycans [33], assembled as a catalytically active head‐to‐tail homodimer [34]. LPL can also associate with GPIHBP1, which translocates LPL to the capillary wall [29]. The binding of LPL to GPIHBP1 can allow for its bi‐directional transport across the capillary wall to facilitate lipolysis in the capillary lumen [29].

### LPL catalytic and noncatalytic activities

While LPL can hydrolyze fatty acyl chains from triacylglycerols and phospholipids, it exhibits a preference for triacylglycerols over phospholipids [35]. In line with this, LPL preferentially hydrolyzes lipids from triacylglycerol‐rich lipoproteins, including chylomicrons and very low‐density lipoproteins (VLDL) [36]. The metabolism of these lipoproteins will yield chylomicron remnants and intermediate‐density lipoproteins, which can be further metabolized by hepatic lipase and endothelial lipase. The localized LPL activity at cell surfaces *in vivo* is not normally detected systemically without the displacement of LPL via heparin. The catalytic activity of LPL is strongly enhanced by the cofactor apolipoprotein C‐II, while it can be inhibited by apolipoprotein C‐III [37].

In addition to its catalytic function, LPL plays a noncatalytic role in enhancing the catabolism of intact lipoproteins. Through its binding to cell surface proteoglycans, LPL can ‘bridge’ lipoproteins to cell surface receptors, such as select members of the low‐density lipoprotein receptor family, to allow for receptor‐mediated endocytosis. For example, Lupien *et al*. [38] showed in a time‐ and dose‐dependent manner with the MDA‐MB‐231 triple‐negative breast cancer cell line that VLDL could be endocytosed by the VLDL receptor through this bridging function. Thus, this noncatalytic role of LPL, together with its catalytic activity, could provide additional lipids in bulk within the breast tumor environment.

### Meta‐analyses of *LPL* mRNA expression in breast tumors

**Fig. 2:**
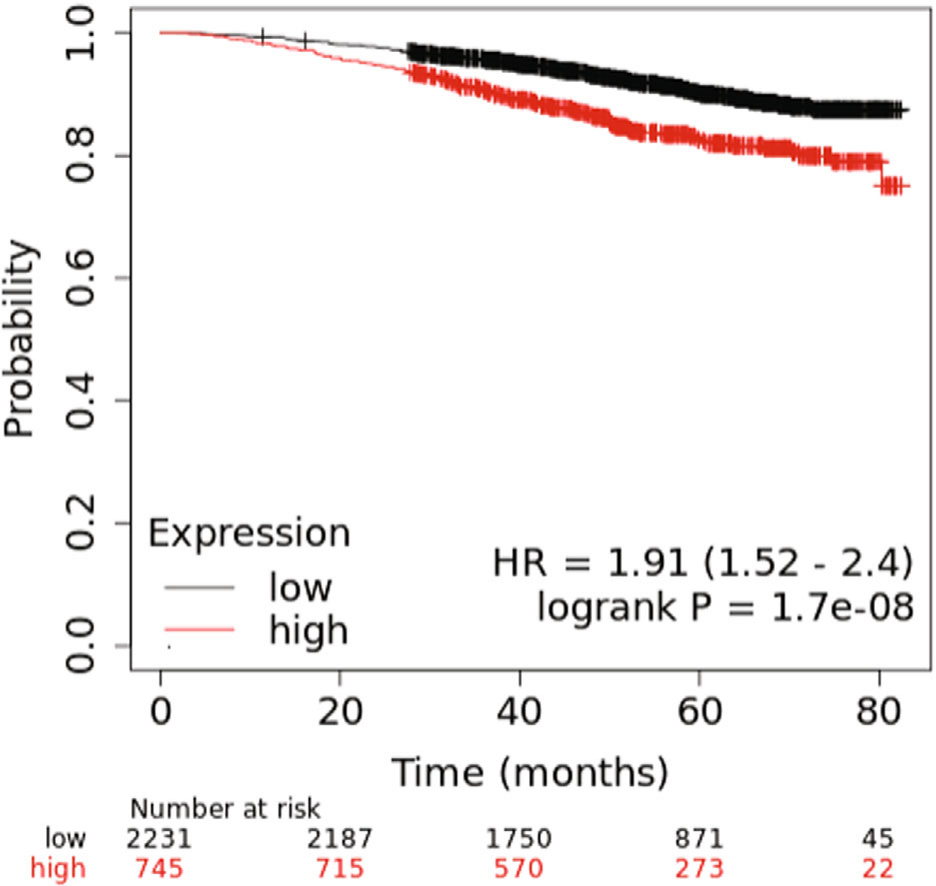
Kaplan–Meier plot of the probability of survival over time for subjects with high or low expression of *LPL* mRNA within breast tumors assessed by RNAseq. RNAseq datasets for all types of breast cancer tumors primarily from The Cancer Genome Atlas database were examined for *LPL* mRNA expression, using kmplot online software (http://www.kmplot.com/) [13]. Subjects were defined as high expressors or low expressors of *LPL* mRNA based on the automatic calculation of the upper and lower quartiles of the arbitrary values of RNAseq data. Hazard ratios and statistical analyses were calculated with the online software, as previously described [13].

In 2011, Kuemmerle et al. [12] reported that LPL protein was present in 147 examined breast tumors. This would support the need for LPL by the tumor environment to provide fatty acids. An examination of RNAseq data (primarily from The Cancer Genome Atlas database) via the KMPlot analysis tool for breast tumor *LPL* mRNA expression revealed that a lower rate of survival over 80 months was associated with a high expression of *LPL*, regardless of breast cancer subtype—2976 subjects, HR 1.91 (1.52, 2.40), *P* = 1.70 × 10^−8^ (Fig. 2 and Table S1). The luminal B ER^+^/PR^−^/HER2^+^ subtype [*n* = 40, HR 15.00 (1.63, 137.82), *P* = 0.0023] and the HER2‐enriched ER^−^/PR^−^/HER2^+^ subtype [*n* = 50, HR 6.07 × 10^8^ (0, infinity), *P* = 0.043] appear to in part contribute to the association of high *LPL* mRNA expression and low survival rate with breast cancer (Table S1). A contradictory observation was found with the luminal A ER^−^/PR^+^/HER2^−^ subtype, such that a high level of *LPL* mRNA expression was associated with a higher survival rate [*n* = 21, HR 0.00 (0.00, infinity), *P* = 0.039] (Table S1). No differences were observed with other breast cancer subtypes (Table S1). Some caution with the interpretation of the aforementioned data is needed, in part due to a low number of subjects for some subtypes, and also because an examination of combined microarray datasets from the Gene Expression Omnibus and European Genome‐Phenome Archive databases via the KMPlot analysis tool showed no significant differences between *LPL* mRNA expression and survival (Table S2). This is likely due to differences in microarray and RNAseq methodologies; however, more reliability may be placed on the RNAseq data because of the ability to read longer sequences compared with the probe lengths in DNA microarrays [39].

Given the above meta‐data examining tumor tissue, it would be anticipated that the ER^+^/PR^−^/HER2^+^ and ER^−^/PR^−^/HER2^+^ breast cancer cell lines would express more *LPL* mRNA versus other breast cancer cell subtypes. Kuemmerle et al. [12] evaluated *LPL* mRNA expression in several breast cancer cell lines via qPCR. They showed that the luminal B ER^+^/PR^−^/HER2^+^ BT474 cell line and the HER2‐enriched ER^−^/PR^−^/HER2^+^ SKBR3 cell line had among the lowest levels of *LPL* mRNA versus other breast cancer cell subtypes. This discrepancy highlights the complexity of tumor tissue, which aside from the breast cancer cells themselves, contains surrounding adipose tissue and endothelial vasculature that also expresses *LPL* mRNA and active LPL protein (as well as other lipases). Thus, additional cell types in the tumor microenvironment may provide a source of the enzyme to yield fatty acids from acylglycerols for use by breast cancer cells.

**Fig. 3:**
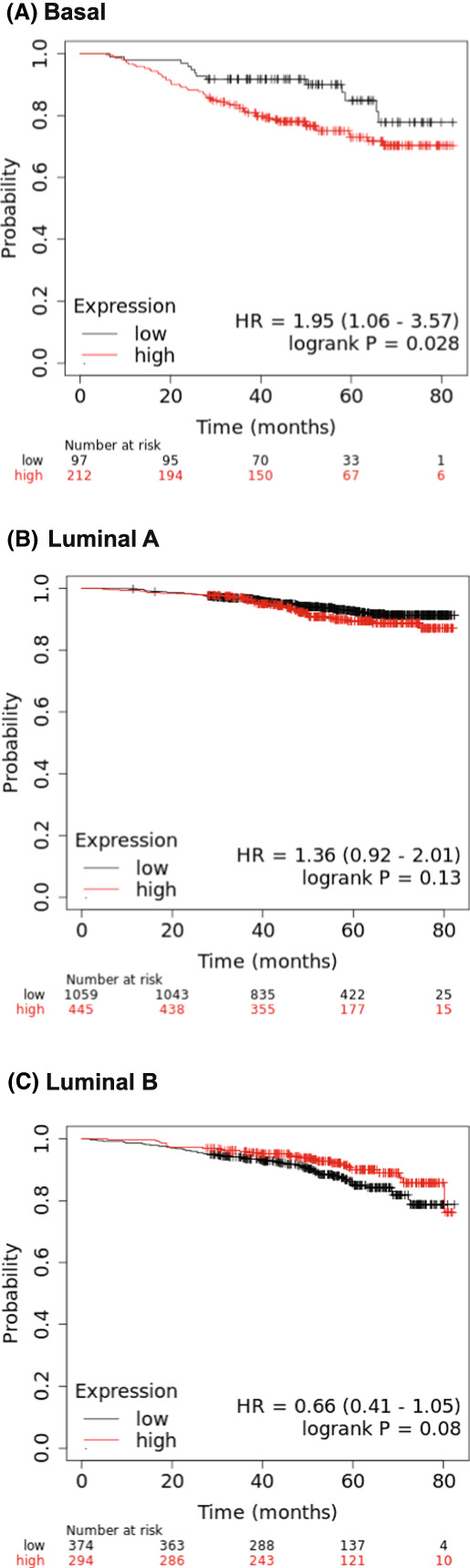
Kaplan–Meier plots of the probability of survival over time for subjects with high or low expression of *LPL* mRNA within basal and luminal breast tumors assessed by RNAseq. RNAseq datasets from The Cancer Genome Atlas database for (A) basal‐type tumors, (B) luminal A‐type tumors, and (C) luminal B‐type tumors were examined for *LPL* mRNA expression, using kmplot online software (http://www.kmplot.com/) [13]. Subjects were defined as high expressors or low expressors of *LPL* mRNA based on the automatic calculation of the upper and lower quartiles of the arbitrary values of RNAseq data. Hazard ratios and statistical analyses were calculated with the online software, as previously described [13].

Kuemmerle et al. [12] showed that the triple negative ER^−^/PR^−^/HER2^−^ cell line Du4475 had the highest level of *LPL* mRNA expression compared with all other breast cancer cell subtypes examined. LPL protein was shown on these cells by immunoblotting [12] and subsequently by flow cytometry [38], with the LPL being catalytically active [12]. Kuemmerle et al. [12] showed five other cell lines exhibited the highest level of *LPL* mRNA expression, but their receptor statuses were somewhat inconsistent: HCC2157 (ER^−^/PR^+^/HER2^+^), HCC1008 (ER^−^/PR^−^/HER2^+^), HCC1599 (ER^−^/PR^−^/HER2^−^), SUM149 (ER^−^/PR^−^/HER2^−^), and SUM190 (ER^−^/PR^−^/HER2^−^). Despite this, these five cell lines plus the Du4475 cell line were considered aggressive basal‐type cells. A re‐examination of datasets for RNAseq via the KMPlot analysis tool showed that basal‐type tumors indeed had a reduced survival rate with high *LPL* mRNA expression [*n* = 309, HR 1.95 (1.06, 3.57), *P* = 0.028], unlike both luminal A‐type tumors [*n* = 1504, HR 1.36 (0.92, 2.01), *P* = 0.13] and luminal B‐type tumors [*n* = 668, HR 0.66 (0.41, 1.05), *P* = 0.080], both of which exhibited no association (Fig. 3 and Table S3).

### LPL protein and *LMF1* mRNA in breast cancer

**Fig. 4:**
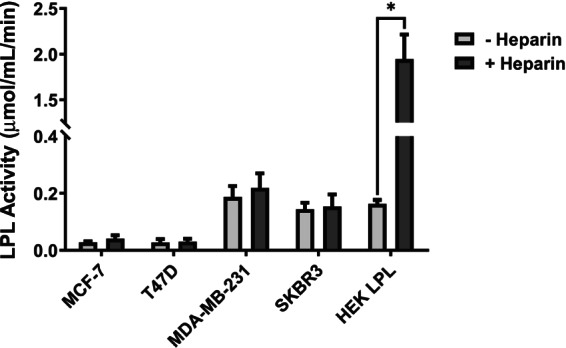
LPL activity from the media of breast tumor cell lines in the absence or presence of heparin. Breast cancer cell lines were incubated for 30 min. in the absence or presence of heparin within serum‐free media. Heparinized media from HEK‐293T cells transiently transfected to express LPL were used as a positive control. Media were examined for LPL activity using a resorufin ester substrate. Data are presented as the mean of triplicate experiments ± standard deviation. *P < 0.05 using an unpaired *t*‐test. Experimental design and details are available within the Materials and methods.

Using flow cytometry, Lupien et al. [38] reported the presence of LPL protein associated with the BT‐474 (ER^+^/PR^−^/HER2^+^) and the MDA‐MD‐231 (ER^−^/PR^−^/HER2^−^) cell lines, although almost all of the LPL protein was detected intracellularly. Other cell lines, such as the T47D (ER^+^/PR^+^/HER2^−^) and MCF‐7 (ER^+^/PR^+^/HER2^−^) lines, exhibited little *LPL* mRNA expression [12] and no detectable endogenous LPL protein [38]. Consistent with these data, we detected no LPL activity in both the absence or presence of heparin from the media of T47D and MCF‐7 cells (Fig. 4). However, we did detect a low level of LPL activity from the media of MDA‐MD‐231 and SKBR3 cells, although comparable in the absence or presence of heparin (Fig. 4).

**Fig. 5:**
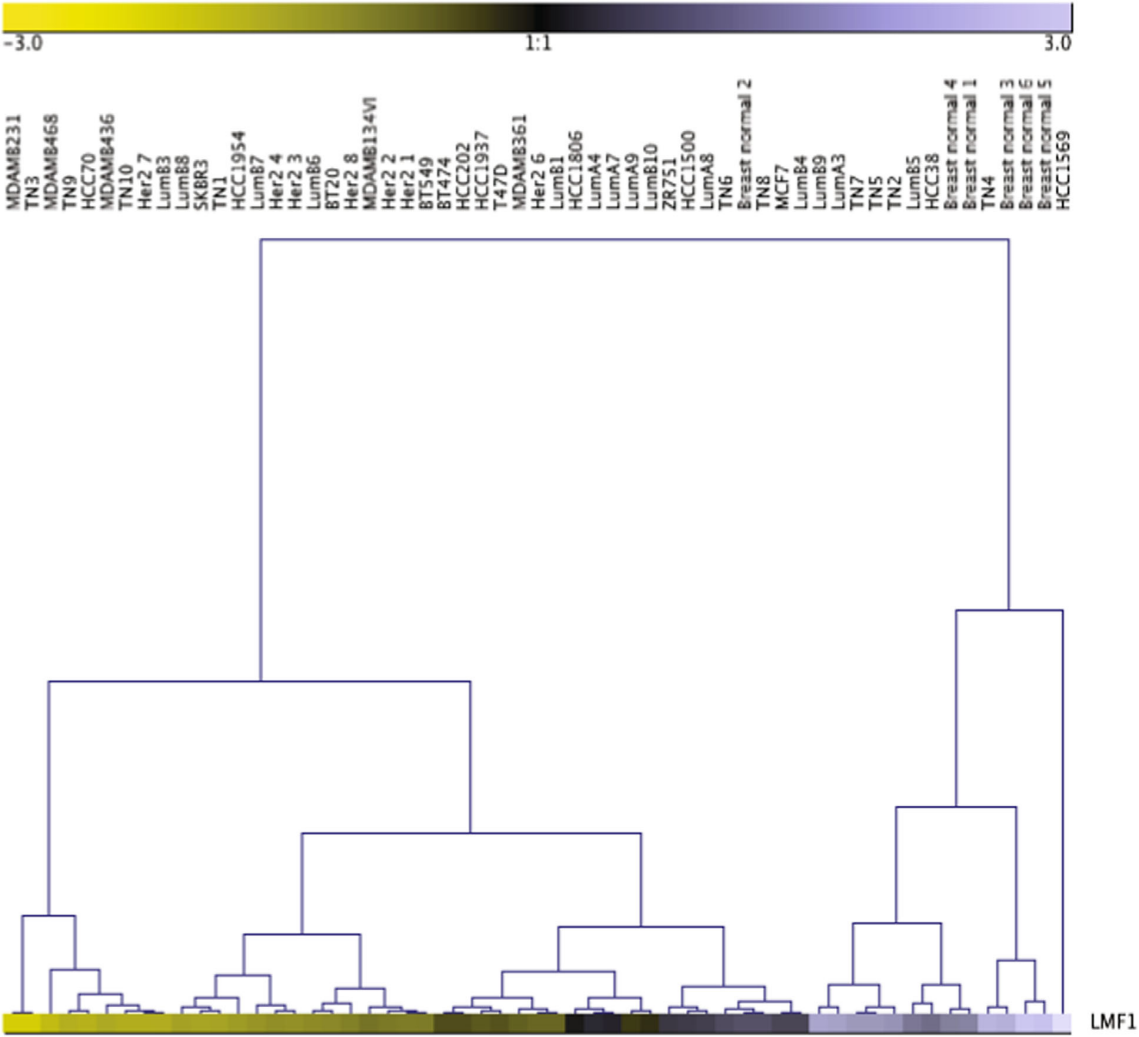
Analysis of *LMF1* mRNA expression in breast cancer cell lines, primary breast tumor samples, and normal breast tissue. Gene expression data from 20 breast cancer cell lines, 31 breast cancer patient samples, and six normal breast tissue samples were obtained from the Gene Expression Omnibus database. *LMF1* probe IDs were obtained and used to filter and average the gene expression data. Gene expression data were analyzed by complete linkage hierarchical clustering using genesis 1.8.1 [19]. Details of the analysis are available within the Materials and methods.

The intracellular accumulation of LPL protein observed by Lupien et al. [38], and our observation of low LPL activities from the MDA‐MD‐231 and SKBR3 cells, may suggest a low expression of the chaperone lipase maturation factor 1 (LMF1) in the breast cancer cell lines; LMF1 is needed to yield active LPL within the secretory pathway [40]. Thus, we examined *LMF1* mRNA expression in 20 breast cancer cell lines, 31 breast cancer patient samples, and six normal breast tissue samples using datasets obtained from the Gene Expression Omnibus [18]. The results show that across the majority of samples tested, *LMF1* mRNA expression is lower in breast cancer tissues and cell lines versus normal breast tissue (Fig. 5). Thus, the data suggest that LMF1 expression may become dysregulated in breast cancer; however, this requires further investigation.

### What is LPL doing in the breast cancer microenvironment?

The expression of *LPL* mRNA or the presence of LPL protein, whether active or inactive, in breast cancer tumor subtypes or cell types points toward LPL providing fatty acids for use within the tumor environment. The overall question is why is LPL, a key enzyme for lipoprotein metabolism and the prevention of systemic hypertriglyceridemia, present in the tumor microenvironment in the first place? This can be followed by the question of whether the enzyme itself—active or inactive, or the hydrolysis products generated by the enzyme, is contributing toward a beneficial or detrimental outcome in the breast cancer tumor milieu. Data to thoroughly address these questions are very limited. However, the available data open future avenues of research toward understanding the role of LPL in breast cancer, and other cancers in general.

Lupien et al. [38] showed that Di‐I‐labeled VLDL could bind to the cell surfaces of MDA‐MB‐231 cells and that it could be internalized at 37 °C. Heparin, heparinase, and antibodies against heparan sulfate proteoglycans prevented this association and uptake. Furthermore, the authors showed that the siRNA‐mediated knockdown of LPL or the VLDL receptor, as well as the incubation of cells with receptor‐associated protein, also reduced Di‐I‐labeled VLDL uptake, thus suggesting a role for the bridging function of LPL. On the contrary, Kuemmerle et al. [12] showed that Di‐I‐labeled VLDL could bind to the cell surfaces of Du4475 cells, but they did not observe the internalization of the Di‐I‐labeled VLDL. This suggests a possible dysfunction with one or more of the low‐density lipoprotein receptor family members rather than an issue with LPL itself since it was found to be catalytically active. However, this route of lipid delivery in the presence of increasing exogenous LPL was shown to increase cell viability in T47D cells [12]. Thus, both endogenous and exogenous LPL protein appear to be detrimental to breast cancer.

Backing a detrimental role of LPL protein in breast cancer is work by Manupati et al. [41], who showed the activation of the transmembrane glycoprotein CD44 with hyaluronic acid in CD24^−^/CD44^+^ breast cancer stem cells upregulated *LPL* mRNA and LPL protein expression; the siRNA‐mediated knockdown of CD44 reduced LPL protein expression versus control cells with scrambled siRNA. The authors further showed *in vitro* that the pan‐*sn*‐1 lipase inhibitor tetrahydrolipstatin significantly inhibited hyaluronic acid‐induced increases in migration, invasion, and mammosphere formation in Matrigel cultures of the CD24^−^/CD44^+^ breast cancer stem cells isolated from MDA‐MD‐231 cells. Lastly, using the immunocompromised nude mouse model, the authors showed that the intratumoral administration of tetrahydrolipstatin to tumors derived from CD24^−^/CD44^+^ breast cancer stem cells expressing green fluorescent protein as a marker for detection resulted in a nearly 20‐fold lower tumor size after 65 days versus untreated CD24^−^/CD44^+^ breast cancer stem cell‐derived tumors. While this suggests LPL as the key player in affecting tumor size, it should be noted that the presence of endothelial lipase may also affect tumor growth [42].

Cancer cells can secrete cytokines to modulate the survival of the cells and the surrounding tumor microenvironment [43]. Our group, in Tobin et al. [14], incubated MDA‐MB‐231, MDA‐MB‐468, MCF‐7, SKBR3, T47D cell lines, and the noncancerous mammary epithelial cell line MCF‐10a, in the absence or presence of lipoprotein lipid hydrolysis products that were generated by LPL. ELISA analyses were carried out for tumor necrosis factor (TNF)‐α, interleukin (IL)‐4, and IL‐6 from the media of the cell lines. Our group found a significant increase in the levels of TNF‐α, IL‐4, and IL‐6 in the media from triple‐negative breast cancer cell lines (MDA‐MB‐231 and MDA‐MB‐468) compared with the media from MCF‐10a control cells, a significant increase in media IL‐6 from SKBR3 cells versus control but no detectable levels of TNF‐α, IL‐4, or IL‐6 within the media from MCF‐7 and T47D cells. TNF‐α has been shown to play a role in cancer cell proliferation, angiogenesis, migration, and invasion, and it is typically associated with more aggressive subtypes [44]. Similarly, IL‐6 also plays a significantly detrimental role in the tumor microenvironment and tumor metastasis [45]. IL‐4 secretion can activate M2‐like tumor‐associated macrophages, which are strongly protumorigenic and can contribute to a protumorigenic microenvironment *in vivo* [46]. Collectively, this indicates that the hydrolysis products liberated by LPL from total lipoproteins can play a detrimental role in breast cancer.

## Conclusion

Although several nuances remain to be studied, LPL has been extensively investigated for its roles in lipoprotein metabolism and atherosclerosis, as noted in many reviews such as Olivecrona and Olivecrona [20], Li et al. [21], and Kumari et al. [22]. However, research toward understanding the roles of LPL in breast cancer (and other cancers) is in its infancy. In addition, a careful examination of the classes and individual species of lipid hydrolysis products generated by LPL from lipoproteins, and their roles in breast cancer progression, remain to be understood. Reducing LPL activity in the tumor microenvironment appears to be an attractive concept [41]. However, a risk of some degree of systemic hypertriglyceridemia might exist that may contribute to atherosclerosis, pancreatitis, and steatosis. Of note, some chemotherapies, such as tamoxifen and asparaginase, inhibit LPL activity but also lead to hypertriglyceridemia [47, 48]. A reduction in LPL activity as part of breast cancer therapy combined with additional therapies to reduce hypertriglyceridemia, such as fenofibrate [49] and high‐dose omega‐3 fatty acids [50], may protect against the hypertriglyceridemia burden; however, this remains to be examined.

**Fig. 6:**
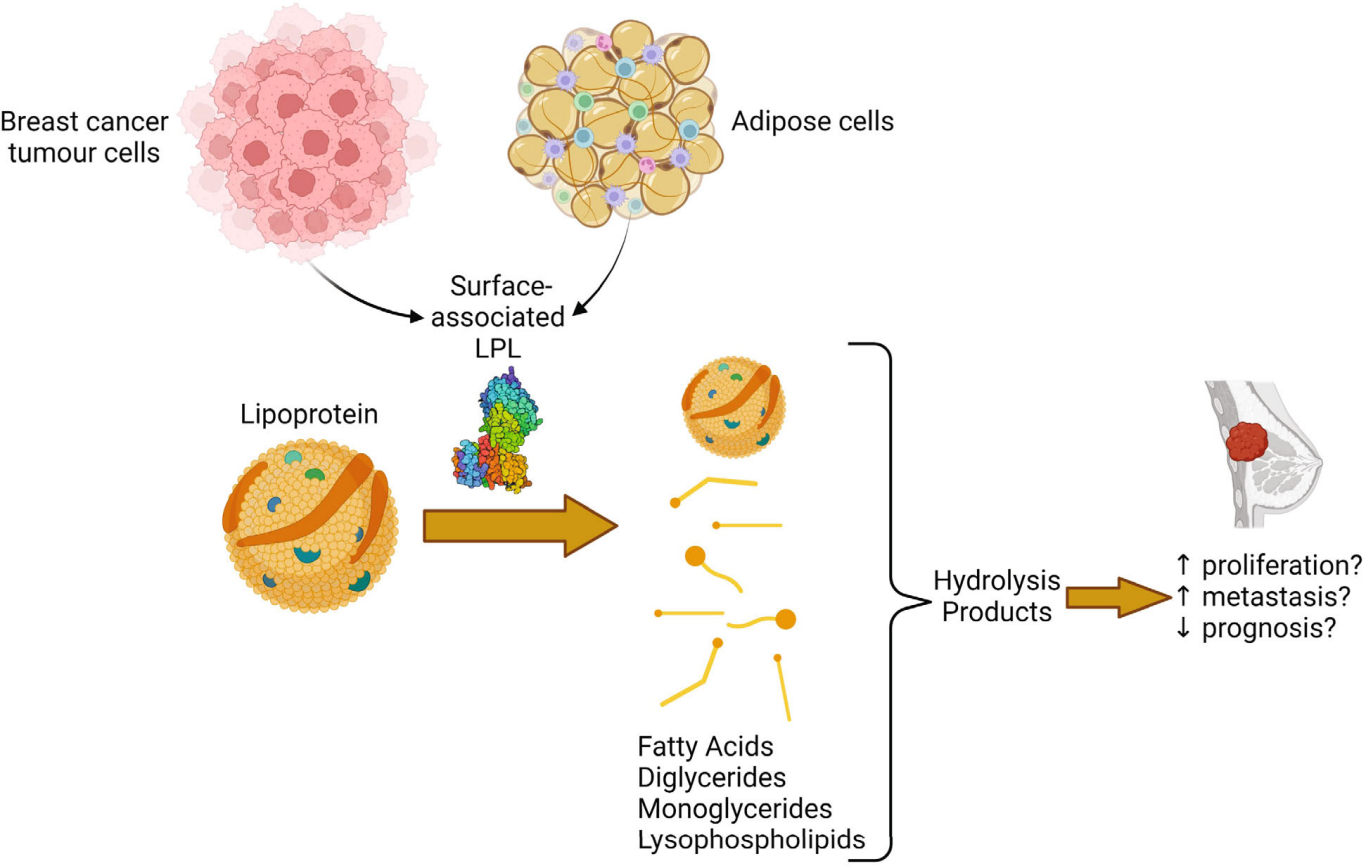
Hypothesis for the role of LPL in the breast cancer microenvironment. Cell surface‐associated LPL, from either breast cancer cells or surrounding adipose tissue, will hydrolyze lipoprotein lipids to yield smaller lipoproteins and lipid hydrolysis products (including unesterified fatty acids, acylglycerides, and lysophospholipids). We hypothesize that these products will result in breast cancer cell proliferation and metastasis, leading to a reduced prognosis.

Overall, we hypothesize that the lipid hydrolysis products that are generated from lipoproteins by LPL impact the breast cancer cell microenvironment, ultimately decreasing prognosis due to increased proliferation and metastasis (Fig. 6). Further studies into the role of LPL in different cancers are necessary to understand the impact LPL has on tumorigenesis, and how it can be used as a potential target for therapy. The role of LPL may vary depending on the stage of cancer and subtype, but the time is right to take advantage of the biochemistry of LPL and the knowledge of LPL function in atherosclerosis down a new avenue.

## Conflict of interest

The authors declare no conflict of interest.

## Author contributions

MMB and RJB conceived the study. MMB, AMN, and AJT acquired the data. MMB, AMN, AJT, SLC, and RJB analyzed and interpreted the data, drafted the manuscript, read the final manuscript, and approved the final manuscript.

## Supporting information


**Table S1.** RNAseq analysis of *LPL* mRNA expression within breast cancer tumors.

**Table S2.** Microarray analysis of *LPL* mRNA expression within breast cancer tumors.

**Table S3.** RNAseq analysis of *LPL* mRNA expression for basal and luminal A/B breast cancer tumor subtypes.

## Data Availability

All RNAseq and microarray datasets used for *LPL* are publicly accessible via the KMplot online analysis tool (http://www.kmplot.com/) [13]. Datasets used to examine *LMF1* were previously reported [35]. Experimental data are available upon request to the corresponding authors.
