# Supplementary material for: The breast cancer microenvironment and lipoprotein lipase: Another negative notch for a beneficial enzyme?
Source: FEBS Open Bio. 2023 Jan 30;13(4):586–96. doi: 10.1002/2211-5463.13559 (PMC10068309; doi:10.1002/2211-5463.13559)
Supplement: Supplementary file 1 — Table S1. RNAseq analysis of LPL mRNA expression within breast cancer tumors. [file FEB4-13-586-s001.docx]

**Supplementary Table S1: RNAseq analysis of *LPL* mRNA expression within breast cancer tumors**

| **Subtype** | # Subjects | Hazard Ratio & Range | P value |
| --- | --- | --- | --- |
|  |  |  |  |
| All subtypes | 2,976 | 1.91 (1.52, 2.40) | 1.70 × 10^-8^ |
| ER+/PR+/HER2+ | 203 | 2.12 (0.88, 5.14) | 0.088 |
| ER+/PR+/HER2- | 2,005 | 0.75 (0.55, 1.02) | 0.067 |
| ER+/PR-/HER2+  ER+/PR-/HER2-  ER-/PR+/HER2+  ER-/PR+/HER2-  ER-/PR-/HER2+  ER-/PR-/HER2- | 40  118  6  21  50  126 | 15.00 (1.63, 137.82)  2.32 (0.96, 5.61)  ND   1. (0, infinity)   6.07 × 10^8^ (0, infinity)  2.26 (0.85, 6.03) | 0.0023  0.056  ND  0.039  0.043  0.094 |
