# Supplementary material for: The breast cancer microenvironment and lipoprotein lipase: Another negative notch for a beneficial enzyme?
Source: FEBS Open Bio. 2023 Jan 30;13(4):586–96. doi: 10.1002/2211-5463.13559 (PMC10068309; doi:10.1002/2211-5463.13559)
Supplement: Supplementary file 2 — Table S2. Microarray analysis of LPL mRNA expression within breast cancer tumors. [file FEB4-13-586-s003.docx]

**Supplementary Table S2: Microarray analysis of *LPL* mRNA expression within breast cancer tumors**

| **Subtype** | # Subjects | Hazard Ratio & Range | P value |
| --- | --- | --- | --- |
|  |  |  |  |
| All subtypes | 4,929 | 0.94 (0.85, 1.04) | 0.20 |
| ER+/PR+/HER2+ | 100 | 1.20 (0.56, 2.60) | 0.64 |
| ER+/PR+/HER2- | 754 | 1.17 (0.84, 1.63) | 0.36 |
| ER+/PR-/HER2+  ER+/PR-/HER2-  ER-/PR+/HER2+  ER-/PR+/HER2-  ER-/PR-/HER2+  ER-/PR-/HER2- | 34  200  3  19  95  392 | 1.40 (0.43, 4.59)  1.10 (0.63, 1.90)  ND  ND  1.50 (0.79, 2.84)  0.91 (0.64, 1.31) | 0.58  0.74  ND  ND  0.21  0.62 |
