# Supplementary material for: The breast cancer microenvironment and lipoprotein lipase: Another negative notch for a beneficial enzyme?
Source: FEBS Open Bio. 2023 Jan 30;13(4):586–96. doi: 10.1002/2211-5463.13559 (PMC10068309; doi:10.1002/2211-5463.13559)
Supplement: Supplementary file 3 — Table S3. RNAseq analysis of LPL mRNA expression for basal and luminal A/B breast cancer tumor subtypes. [file FEB4-13-586-s002.docx]

**Supplementary Table S3: RNAseq analysis of *LPL* mRNA expression for basal and luminal A/B breast cancer tumors subtypes**

| **Subtype** | # Subjects | Hazard Ratio & Range | P value |
| --- | --- | --- | --- |
|  |  |  |  |
| Basal | 309 | 1.95 (1.06, 3.57) | 0.028 |
| Luminal A | 1,504 | 1.36 (0.92, 2.01) | 0.13 |
| Luminal B | 668 | 0.66 (0.41, 1.05) | 0.080 |
